# Maternal immunization with ovalbumin prevents neonatal allergy development and up-regulates inhibitory receptor FcγRIIB expression on B cells

**DOI:** 10.1186/1471-2172-11-11

**Published:** 2010-03-11

**Authors:** Jefferson R Victor, Bruno P Muniz, Ana E Fusaro, Cyro A de Brito, Eliana F Taniguchi, Alberto JS Duarte, Maria N Sato

**Affiliations:** 1 Laboratory of Dermatology and Immunodeficiencies, School of Medicine, University of São Paulo, LIM 56, São Paulo, Brazil

## Abstract

**Background:**

Preconception allergen immunization prevents neonatal allergen sensitization in mice by a complex interaction between regulatory cells/factors and antibodies. The present study assessed the influence of maternal immunization with ovalbumin (OVA) on the immune response of 3 day-old and 3 week-old offspring immunized or non-immunized with OVA and evaluated the effect of IgG treatment during fetal development or neonatal period.

**Results:**

Maternal immunization with OVA showed increased levels of FcγRIIb expression in splenic B cells of neonates, which were maintained for up to 3 weeks and not affected by additional postnatal OVA immunization. Maternal immunization also exerted a down-modulatory effect on both IL-4 and IFN-γ-secreting T cells and IL-4 and IL-12- secreting B cells. Furthermore, immunized neonates from immunized mothers showed a marked inhibition of antigen-specifc IgE Ab production and lowered Th2/Th1 cytokine levels, whereas displaying enhanced FcγRIIb expression on B cells. These offspring also showed reduced antigen-specific proliferative response and lowered B cell responsiveness. Moreover, *in vitro *evaluation revealed an impairment of B cell activation upon engagement of B cell antigen receptor by IgG from OVA-immunized mice. Finally, *in vivo *IgG transference during pregnancy or breastfeeding revealed that maternal Ab transference was able to increase regulatory cytokines, such as IL-10, in the prenatal stage; yet only the postnatal treatment prevented neonatal sensitization. None of the IgG treatments induced immunological changes in the offspring, as it was observed for those from OVA-immunized mothers.

**Conclusion:**

Maternal immunization upregulates the inhibitory FcγRIIb expression on offspring B cells, avoiding skewed Th2 response and development of allergy. These findings contribute to the advancement of prophylactic strategies to prevent allergic diseases in early life.

## Background

Several studies with mouse or rat models have demonstrated that maternal immunization can suppress specific IgE Ab response in the offspring [1-10]. Targeting the maternal immune system is an attractive strategy for controlling early neonatal allergen sensitization, when infants with pronounced Th2 responses are susceptible to allergic diseases [11,12].

It has been shown that preconception immunization of female mice with the dust mite *Dermatophagoides pteronyssinus *(Der p) transfers high titers of antibodies through the transamniotic/transplacental route and TGF-β-enriched milk by breast feeding [7], leading to the inhibition of both allergen-specific IgE Ab and Th2 cytokine production [9]. The efficacy of maternal immunization was confirmed by the ability to prevent neonatal allergen sensitization when mothers were intensively exposed to Ag during the breastfeeding period [8]. Moreover, breastfeeding-induced tolerance, associated with the presence of TGF-β during lactation, seems to be mediated by regulatory CD4+ T lymphocytes and dependent on the TGF-β signaling in T cells, but does not require the transfer of immunoglobulin [13]. In fact, several mechanisms acting synergistically, involving maternal antibodies (MatAb), regulatory T lymphocytes, and factors that are major components in maternal immunomodulation, are required to prevent offspring allergic responses.

Circulating MatAb in the offspring may diminish allergen processing and presentation by antigen-presenting cells (APCs) to T cells, preventing neonatal sensitization [11]. The immune complex of MatAb involving inhaled or ingested allergens could be cleared before priming the neonate immune system, avoiding IgE Ab production. MatAb transferred to the offspring may recognize the idiotype in the B cell antigen receptors (BCRs) or T cell antigen receptors (TCRs) of immature fetal B or T cells, respectively, interfering with the idiotype repertoire selection [14,15] or, through anti-idiotype interaction with BCRs, promoting a long-lasting inhibitory effect [16,17]. Furthermore, immune complex of MatAb engage BCRs with the IgG receptor on B cells (Fcγ RIIB), delivering a potent inhibitory signal that prevents B cells proliferation and Ab secretion [18]. Nonetheless, so far, there has been no evidence in allergy related studies to suggest that MatAb affect the activation of inhibitory signals through FcγRIIb in neonatal B cells.

In the present work, the impact of preconception immunization with ovalbumin (OVA) on the B and T cell function in neonates or lactating mice was assessed. Also, B and T cell responses were evaluated after IgG injections in pregnant mice or in neonates.

## Results

### Up-regulation of FcγRIIb on B cells of offspring from mothers subjected to preconception immunization with OVA

Mouse mothers in the prenatal stage were immunized with OVA and the immunization effect on their offspring was evaluated by measuring immune response-B cells in particular-in 3 day-old neonates and, later, during the weaning period (3 weeks old). The absolute number of splenic B cells (B220+IgM+) of neonates (3 d-o) from immunized mother (1.36 × 10^6 ^cells ± 0.12) was similar to those from nonimmunized mothers (1.06 × 10^6 ^cells ± 0.11). After neonatal immunization, it was observed an increase in the absolute number of splenic B cells in the 20 d-o offspring from immunized mothers (42.04 × 10^6 ^cells ± 3.58) as compared to their counterparts from nonimmunized mothers (31.13 × 10^6 ^cells ± 1.23).

**Figure 1:**
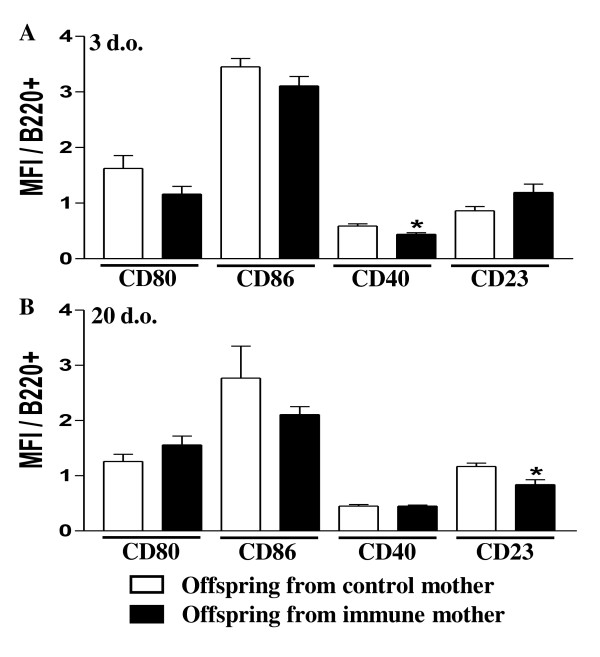
**Influence of maternal immunization with OVA on the *ex vivo *expression of B cell-costimulatory molecules from nonimmunized offspring**. BALB/c offspring from mothers immunized with OVA prior to conception were evaluated at 3 d-o (A) or 20 d-o (B) for CD80, CD86, CD40 and CD23 expression on splenic B cells (B220+IgM+). The data obtained by flow cytometry represent the mean ± SEM of 12 mice per group. * P ≤ 0.05 compared to offspring from nonimmunized mothers.

Figure 1a shows that maternal immunization with OVA induced slight changes in the activation molecule expression in B cells in neonate mice, such as a diminished expression of CD40 compared to the control group; in the 20 day-old group from immunized mothers, only CD23 expression appeared to be altered as compared to the control group (Figure 1b).

**Figure 2:**
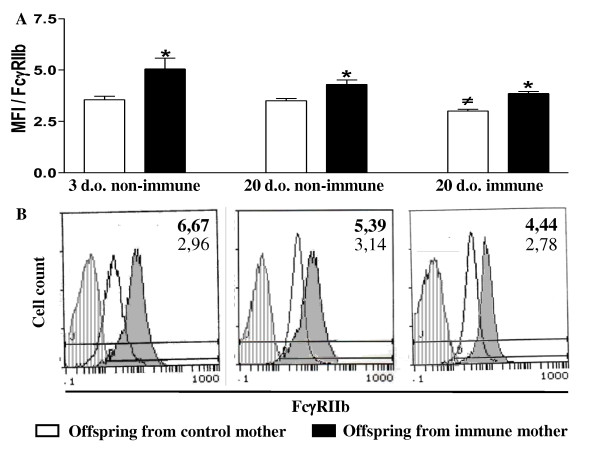
**Maternal immunization with OVA upregulates FcγRIIb expression on B cells from offspring**. BALB/c offspring from mothers immunized with OVA prior to conception or from control mothers (nonimmunized) were immunized (3 d-o) or not with OVA and evaluated at 3 d-o and 20 d-o for FcγRIIb expression on splenic B cells (B220+IgM+). The data obtained by flow cytometry represent the mean ± SEM of 12 mice per group. Histogram of B cell FcγRIIb expression depicting the cells from offspring from immunized (shaded histogram, Mean fluorescence intensity (MFI) in bold numbers) and nonimmunized mothers (white histogram, MFI in light numbers). * P ≤ 0.05 compared to offspring from nonimmunized mothers, # *P *≤ 0.05 compared to nonimmunized offspring (20 d-o) from control mothers.

Neonatal B cells of 3 d-o offspring from immunized mothers showed an increased expression of the inhibitory receptor, FcγRIIb, which was then maintained for 3 weeks, whether the offspring was subjected to neonatal OVA immunization or not (Figure 2).

**Figure 3:**
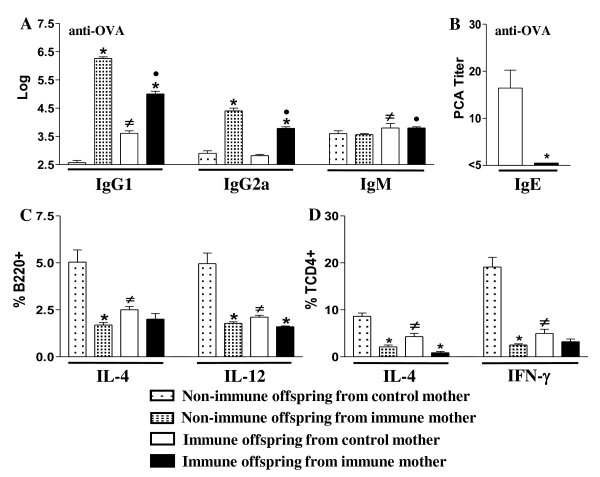
**Effect of maternal immunization with OVA on the immune response of nonimmunized or immunized neonates**. Neonate pups (3 d-o) from control or immune mothers were immunized or not with OVA and evaluated (20 d-o) for: (A) IgG1, IgG2a and IgM by ELISA; (B) anti-OVA IgE Ab levels by PCA reaction; (C) intracellular cytokines of splenic B cells (B220+) or (d) CD4+ T cells after 24 h incubation with 10 μg/mL brefeldin A by flow cytometry. The results represent the mean ± SEM of 12 mice per group. *P ≤ 0.05 compared to offspring from nonimmunized mothers, # *P *≤ 0.05 compared to nonimmunized offspring from control mothers, • *P *≤ 0.05 compared to control offspring from immune mothers.

Maternal immunization correlated with high levels of anti-OVA IgG1 and IgG2a Ab in the pups, and when this offspring was submitted to neonatal immunization, both IgG subclasses were inhibited (Figure 3a). The levels of IgG Ab detected in the immunized offspring represent both the vertically transmitted from the mothers and the offspring's own production [9]. The decrease in the IgG1 and IgG2a Ab levels of immunized offspring from immune mothers indicates that MatAb down-modulate offspring Ab production. The absence of IgM in the offspring, showing no sensitization, suggests that there had been no allergen transfer from mothers. Induction of anti-OVA IgM production was only observed after neonatal immunization. Preconception immunization with OVA significantly diminished anti-OVA IgE Ab production in the immunized offspring (Figure 3b). Furthermore, maternal immunization decreased the percentage of splenic cytokine-secreting B cells (IL-4 and IL-12) and CD4+ T cells (IL-4 and IFN-γ) in the nonimmunized offspring, as compared to the control group (Figure 3c and 3d).

**Figure 4:**
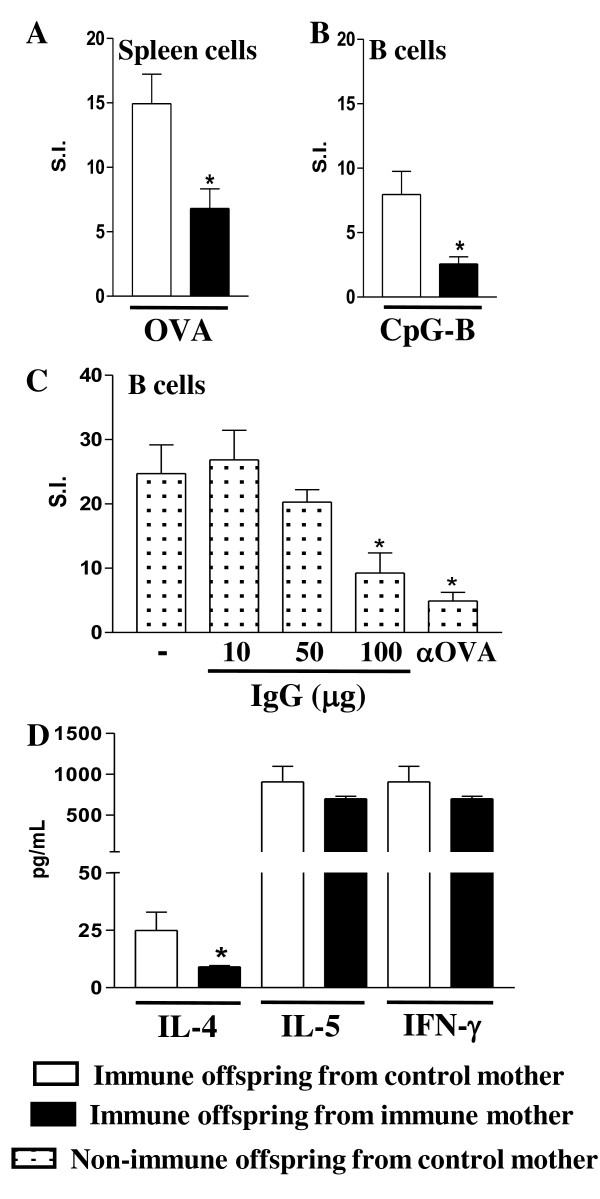
**Impaired B cell activation by BCR-crosslinking of offpring from immunized mothers**. Neonate pups (3 d-o) were immunized with OVA and evaluated (20 d-o) for: (A) proliferative spleen cell response to OVA; (B) purified B-cell response for CpG oligodeoxynucleotide type B (5 μg/mL) stimuli; (C) purified B cell from nonimmunized mice incubated with F(ab')_2 _anti-mouse IgM Ab (50 μg/mL) in the presence of IgG from from OVA-immunized unrelated adult mice (10, 50, 100 μg/mL), or monoclonal antibody anti-OVA (α OVA, 5 μg/mL) plus OVA (10 μg/mL), incubated for 96 h [data expressed in Stimulation Index (S.I.)]; (D) cytokine measurements in the supernatants of spleen cell culture after 72 h of stimulation with OVA by cytometric bead array. The results represent the mean ± SEM of 9 mice per group. *P ≤ 0.05 compared to offspring from nonimmunized mothers.

Neonatal immunization with OVA led to a decreased number of IL-4 and IL-12- secreting B cells and IL-4 and IFN-γ- secreting CD4+ T cells in the offspring from control mothers. Moreover, immunized offspring from immune mothers showed an even lower percentage of IL-12-secreting B cells and IL-4- secreting CD4+ T cells (Figure 3). These findings reveal that early sensitization to OVA is immunomodulatory in pups from both immune and non-immune mothers compared to non-immunizedcontrols and that this effect is more pronounced in pups from immune mothers. Furthermore, maternal immunization significantly lowered the offspring Ag-specific proliferative response (Figure 4a) and B cell responsiveness to CpG stimulus as compared to the control group (Figure 4b). Also, to evaluate whether up-regulation of FcγRIIb expression on B cells could be related to the functional inhibition of B cell activation upon BCR engagement, the proliferative response of B cells from non-immunized mice to anti-IgM crosslinking in presence of IgG and OVA was assessed. The results showed that B cell activation by BCR-crosslinking was significantly inhibited in the presence of IgG and OVA complex at the highest IgG concentration (Figure 4c). In addition, a reduction in IL-4 secretion upon OVA stimulation in offspring from immunized mothers was observed (Figure 4d). The latter result suggests that the maternal immunization prevented offspring allergen sensitization by inhibiting the IgE anaphylactic Ab production and down-modulating the Th2 cytokine production, while simultaneously up-regulating FcγRIIb expression on B cells.

### Effect of IgG transference in the gestational or neonatal periods

To reveal the impact mediated by MatAb, per se, on the offspring's B cell function, purified IgG from immunized or nonimmunized mothers was i.v. injected into pregnant or neonate mice.

**Figure 5:**
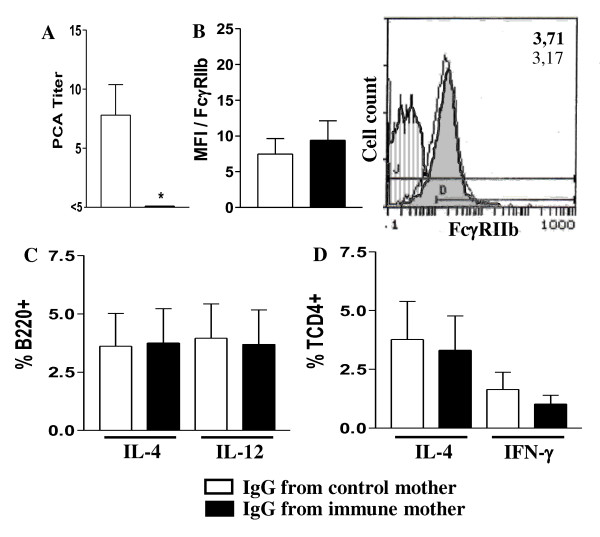
**Effect of passive IgG transference to neonates on B and T cell responses**. Neonate pups (3 d-o) from nonimmunized mothers injected with IgG from nonimmunized or immunized mothers and simultaneously immunized with OVA were evaluated (20 d-o) for: (A) anti-OVA IgE Ab levels by PCA reaction; (B) B cell FcγRIIb expression (B220+IgM+) and histogram of FcγRIIb expression on B cells of offspring from immunized (shaded histogram, MFI in bold numbers) or nonimmunized mothers (white histogram, MFI in light numbers); (C) intracellular cytokines of splenic B cells (B220+) or (D) CD4+ T cells after 24 h incubation with 10 μg/mL brefeldin A; data shown in B-D were obtained flow cytometry. The results represent the mean ± SEM of 9 mice per group. * *P *≤ 0.05 compared to offspring from nonimmunized mothers.

Figure 5 shows that passive IgG transference from immunized mothers to neonates inhibited IgE Ab response compared to the group receiving IgG from nonimmunized mice. However, no changes were observed in the expression of activation/inhibition molecules on B cells or in the intracellular cytokines of B or CD4+ T cells (Figure 5).

To elucidate the effect of MatAb during fetal development, pregnant mice were subjected to i.v. IgG injections on days 10, 15 and 20 of gestation. After delivery, the offspring were evaluated at 3 d-o and at the weaning period, after neonatal immunization.

**Figure 6:**
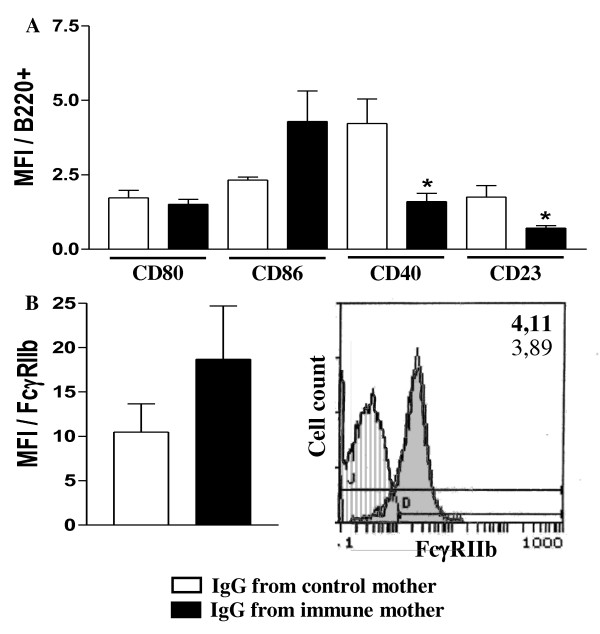
**Effect of passive IgG transference to pregnant mice on neonatal B cell FcγRIIb expression**. Nonimmunized pregnant mice were injected with IgG from nonimmunized or immunized mothers. Nonimmunized neonates were evaluated (3 d-o) for: (A) CD80, CD86, CD40, and CD23 molecule expression on splenic B cells (B220+) and (B) B cell FcγRIIb expression (B220+IgM+) by flow cytometry. Histogram of FcγRIIb expression on B cells of offspring from immunized (shaded histogram, MFI in bold numbers) or nonimmunized mothers (white histogram, MFI in light numbers). The results represent the mean ± SEM of 6 mice per group. * *P *≤ 0.05 compared to offspring from nonimmunized mothers.

**Figure 7:**
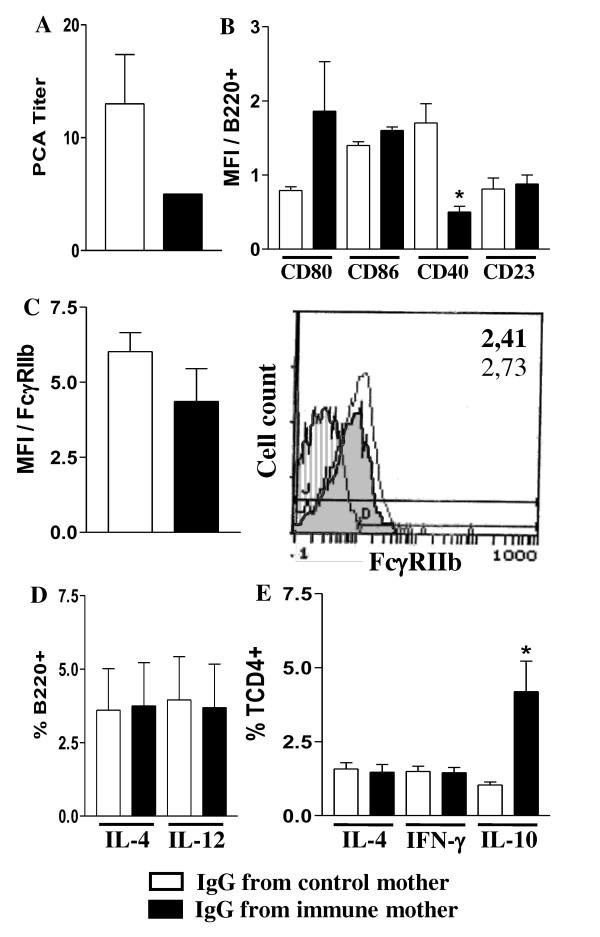
**Effect of passive IgG transference to pregnant mice on offspring's B and T cell responses**. Nonimmunized pregnant mice were injected with IgG from nonimmunized or immunized mothers. Offspring immunized with OVA were evaluated (20 d-o) for: (a) anti-OVA IgE Ab levels by PCA reaction; (b) CD80, CD86, CD40 CD23 molecule expression on splenic B cells (B220+); (c) B cell FcγRIIb expression (B220+IgM+) by flow cytometry. Histogram of FcγRIIb expression on B cells of offspring from immunized (shaded histogram, MFI in bold numbers) or nonimmunized mothers (white histogram, MFI in light numbers); (d) intracellular cytokines of splenic B cells (B220+) and (e) CD4+ T cells after 424 h incubation with 10 μg/mL brefeldin A, all by flow cytometry. The results represent the mean ± SEM of 6 mice per group. *P ≤ 0.05 compared to offspring from nonimmunized mothers.

Non-immunized offspring (3 d-o) from mothers that received IgG from immune mice during pregnancy showed lower expression of CD40 and CD23 molecules on B cells compared to those from pregnant mothers that received non-immune IgG. As for the FcγRIIb expression, the increase observed in pups from immune mothers was not statistically significant (Figure 6). After neonatal immunization, these offspring (20 d-o) showed IgE Ab response and FcγRIIb expression on B cells at similar levels to those from mothers treated with control IgG (Figure 7). Curiously, a high percentage of *ex vivo *IL-10-producing CD4+ T cells was detected in offspring from mothers treated with immunized IgG during pregnancy, with no changes in the IL-4 and IFN-γ cell numbers or in cytokine-secreting B cells.

The results showed that MatAb passively transferred to neonates may prevent IgE Ab response; however, both IgG treatments failed to induce the immunological responses observed in the offspring from OVA-immunized mothers.

## Discussion

Allergy prevention through maternal immunization with specific allergens has been shown to be a promising prophylatic way to avoid sensitization in early life and the development of allergic diseases. The mechanisms underlying the IgE Ab response, as a consequence of maternal immunization, involve a complex interaction of inhibitory MatAb, regulatory cytokines-including TGF-β [8]-and regulatory T cells [13] that are yet to be fully understood. MatAb have a crucial role in forming immune complexes that are able to neutralize allergens and prevent neonatal sensitization. Nonetheless the effect of MatAb through BCR engagement on the B cell function remains unclear.

Our results showed that maternal immunization up-regulates the inhibitory IgG receptor, FcγRIIb, on B cells of neonates at very early age (3 d-o) and in young mice. Furthermore, high levels of Ag-specific IgG Ab were transferred to the pups by transplacental and breastfeeding routes, allowing the crosslinking of Fcγ RIIB through the IgG-Ag complex and leading to the B cell inhibition. The inhibitory coreceptors contain immunoreceptor tyrosine-based inhibition motifs (ITIMs) in the cytoplasmic tails. Phosphorylation of the ITIM of FcγRIIb inhibits *in vitro *activation of B cells [19,20] and immature B cells, which are highly sensitive to Fc*γ*RIIB inhibitory signaling [21]. Also, it has been shown that all B cell stages express FcγRIIB and that crosslinking induces apoptosis of plasma cells, which may help to control their homeostasis [22]. In this work, the functional inhibition of B cell activation upon anti-IgM stimulation in presence of IgG-OVA complex suggests that this interaction up-regulate FcγRIIB expression, leading to the B cell proliferative inhibition. In addition, it has been shown that the inhibitory Fc receptor is also required to maintain tolerance [23]. In lupus-prone mouse strains, partial restoration of FcγRIIB on B cells is sufficient to restore tolerance and prevent autoimmunity [24,25]. Therefore, increasing FcγRIIB levels on B cells may be an effective way to treat autoimmune diseases.

Our data showed that maternal immunization modulates the expression of B cell markers, such as CD23, CD40 and CD44, and reduces the number of IL-12 and IL-4-secreting B cells in nonimmunized offspring. As B cells account for the majority of spleen's cells, they may represent an important cytokine source for CD4+ T cells. Also, considering the role of IL-12 in Th1 activation [26], the diminished number of IL-12-secreting B cells in the offspring from immunized mothers may partially contribute to the reduction in IFN-γ-secreting CD4+ T cells. The control of Th2 function, as verified by the reduced percentage of IL-4-secreting CD4+ T cells and IL-4 secretion, was also down-modulated in offspring from immunized mothers. Therefore, the control of Th1/Th2 cytokine secretion in offspring by maternal immunization seems to be an important strategy to prevent allergen sensitization.

The prophylactic role of maternal immunization was reinforced by neonatal offspring immunization with OVA, as both procedures suppressed the anaphylactic IgE antibodies and allergen-specific proliferative response. The down-modulation of IL-4 production may help to maintain enhanced FcγRIIb expression on B cells in immunized offspring, corroborating to a report showing that IL-4 reduced FcγRIIb-mediated B cell suppression [27]. Indeed, the B cell anergic status in immunized offspring from immunized mothers, hereby observed, was characterized by diminished proliferative responsiveness to CpG oligodeoxynucleotides and suppression of B-cell cytokine secretion. The presence of a TLR-9 agonist response revealed the commitment of other signaling pathways besides BCR's in offspring from immunized mothers. Further investigation is required to ascertain whether signaling via ITIM through FcγRIIB acts as anti-inflammatory by inhibiting NFκB signaling via TLR9 activation. It has been shown that the nonpathogenic immune complex/Ig negatively regulates TLR4-triggered inflammatory response in macrophages, down-regulating NF-κB activation through FcγRIIB-dependent PGE2 [28].

The complex immunological interactions that occur to maintain maternal-fetal tolerance involve many specialized mechanisms to protect the fetus, which expresses paternal Ags, from maternal immune attack [29-32]. Although inbred mouse strains do not evoke aggressive allogeneic responses against the fetus, regulatory mechanisms-like the maternal CD4+CD25+ regulatory T cell pool-are systemically expanded in syngeneic pregnant mice [29]. The mechanisms involved in maternal-fetal tolerance, even in a syngeneic system, may somehow contribute to control the exacerbation of a Th2 response to the allergen. Previously, our group observed that maternal immunization with Der p was able to control the exacerbation of Th2 responses to this allergen in the offspring [9]. In fact, adoptively transferring allergen-specific Th cells to females before mating may cause the offspring to develop asthma [33].

Moreover, maternal adaptive immunity to selective antigens may influences postnatal B cell and antibody responses in offspring [34]. Maternal oxidized LDL immunization before pregnancy induces in offspring an increased IgM Ab to selective OxLDL epitopes, reducing atherosclerosis in offspring. This maternal approach assessed in mice and rabbits point to new strategies to protect offspring against a range of pathogens the mother has become immune, either spontaneously or as result of immunization.

Passive IgG transference was performed to assess the regulatory effect of IgG on the development of fetuses or neonates. We observed that only postnatal IgG injection was able to inhibit offspring IgE Ab response, not interfering with FcγRIIb expression on B cells. Considering that prenatal IgG transference occurs through FcRn, a neonatal IgG Fc receptor [35], independently of Ab specificity, the amount of anti-OVA Ab may not have been enough to neutralize the allergen during offspring immunization, as occurred in offspring treated with IgG at the postnatal stage. Curiously, IgG treatment during pregnancy induced an increased percentage of IL-10-secreting CD4+ T cells after immunization. Moreover, IL-10 is an important regulatory cytokine that can help limit Th1 cytokine production [36] and may represent a regulatory mechanism triggered by the antibodies, leading to idiotypic interactions between TCR and maternal antibodies. In fact, it has been demonstrated that idiotypic interactions between maternal Ab with BCR or TCR during fetal stage can negatively select the B and T lymphocyte repertoire [14,15].

## Conclusions

Our findings showed that the mechanisms involved in the regulation of allergic response by maternal immunization with the allergen ovalbumin are mediated by a complex interaction of regulatory cells/cytokines and antibodies. The MatAb complex alters the progeny immune repertoire through mechanisms that are yet to be fully understood. Nonetheless, there are sufficient and compelling data to justify the research and development of new protocols based on maternal vaccination to prevent allergic diseases.

## Methods

### Animals

BALB/c mice of both sexes (8-10 weeks-old) were obtained from the animal facilities of the São Paulo University Medicine School. Wistar Furth rats of both sexes, 3-4 months-old and bred in our own laboratory's animal facilities, were used for passive cutaneous anaphylaxis (PCA) reaction studies. All the experiments were approved by the Ethics Committee for Animal Research of the Institute of Biomedical Sciences.

### Experimental Protocols

#### Preconception immunization

Female BALB/c mice were immunized s.c. with 150 μg ovalbumin (OVA, grade V, Sigma-Aldrich, St Louis, MO) in 6 mg Al(OH)3 and i.p. boosted with 100 μg OVA without adjuvant, on days 10 and 20 after immunization, as described previously [8]. One day later, the females were mated with nonimmunized male BALB/c mice.

#### Offspring immunization

Three day-old mice of both sexes were i.p. immunized with 10 μg OVA in 0.6 mgAl(OH)3, as described previously [8]. Ten days later, the offspring received an i.p. injection of 10 μg of OVA in saline solution and were bled after 7 days.

### Passive prenatal or postnatal IgG transference

IgG antibodies from sera of mice immunized with OVA (40 days after immunization) or nonimmunized mice were purified using Melon Gel IgG Spin Purification kit, according to the manufacturer's instructions (Pierce, Rockford, IL), and stored at -70°C until use. IgG measurements were performed by ELISA. Prenatal IgG transference was performed in pregnant females by i.v. route with 200 μg of IgG on days 10, 15 and 20 of gestation. Postnatal IgG transference was performed on offspring at 2, 5, 10 and 15 days-old by i.p. route with 10, 30, 60 and 60 μg of IgG, respectively. The non-immunized offspring were assessed at 3 days-old or, when submitted to neonatal immunization with OVA (3 d-o), at 20 d-o.

### Passive cutaneous anaphylaxis (PCA)

IgE antibodies were estimated by PCA in rats according to Mota and Wong [37]. Serum dilutions were inoculated intradermally (100 μL) on the shaved backs of rats. After 18 h, the rats received an injection of 0.5 mg OVA in 1.0 mL of 0.5% Evans Blue solution through a tail vein. PCA titers were expressed as the reciprocal of the highest dilution that caused a spot larger than 5 mm in diameter.

### Determination of Ab levels

OVA-specific IgG1, IgG2a and IgM antibodies were measured by ELISA, as previously described [8]. The results were expressed as antibodies titers with reference to serial dilution of a titrated serum pool from immunized adult mice with high levels of specific Abs.

### Proliferation assay with tritiated thymidine

Spleen aseptically collected from 20 day-old mice was pressed through a cell strainer (BD Biosciences, Bedford, MA) in RPMI-1640 supplemented with 10% FCS (Hyclone III, Lotan, CT). The red blood cells were lysed using ACK Lysing Buffer (Biosource, Rockville, MD) for 90 sec. Resting B cells were purified from splenic mononuclear cells (SMC) using magnetic microbeads from a B cell isolation kit (Miltenyi Biotec, CA, EUA), and enrichment was more than 95% when verified by flow cytometry.

Cultures of SMC (2.0 × 10^5 ^cells/0.2 mL) in 96-well microplates (Costar, Cambridge, MA, UK) were stimulated with OVA (200 μg/mL; Sigma) at 37°C in a humidified 5% CO_2 _incubator. B cell cultures (5 × 10^5 ^cells/0.2 mL) were incubated with 5 μg/mL of CpG olideoxynucleotide (ODN) type B (1826 - 5' TCC ATG ACG TTC CTG ACG TT 3' synthetized by Eurogentec, Belgium). Other B cell cultures (8 × 10^5 ^cells/0.2 mL) were incubated with 50 μg/mL of F(ab')2 goat anti-mouse IgM (Southern Biotechnology Ass., Birmingham, AL) and concentrations of purified IgG (10-100 μg/mL) from immunized mice with OVA or 5 μg/mL of mouse monoclonal to ovalbumin (Abcam Inc, Cambridge, MA) and 10 μg/mL of OVA (Sigma). Thymidine incorporation was measured on day 4 of culture after 18 h of being pulsed with 1 μCi [^3^H]thymidine (Amersham Biosciences AB, Uppsala, Sweden).

### *In vitro *cytokine production

Splenic mononuclear cells were cultured in 48-well plates (Costar) in RPMI-1640 supplemented with 10% FCS with OVA (200 μg/mL, Sigma) for 72 h; the cell-free supernatants were stored at -70°C. Cytokines were measured using a Th1/Th2 cytokine bead array kit (Becton Dickinson, San Diego, CA, USA), by flow cytometry (FACSCAlibur, BD, San Jose, CA).

### Flow cytometry

To evaluate surface markers on SMCs the following mAbs were used: PerCy P-conjugated anti B220, anti-CD4, FITC- labeled anti-IgM (Southern Biotech. Ass., Birmingham, AL), R-PE-conjugated anti-CD40, anti-CD80, anti-CD86, anti-CD23 and anti-CD16/32 (FcγRIII/II) from BD-Pharmingen. All flow cytometry staining procedures were performed at 4°C in PBS/1% BSA (Sigma). Cells were then washed in PBS/1% BSA and flow cytometry buffer before analysis of 10,000 gated events by Coulter Epics XL-MCL (Beckman-Coulter, Miami, FL, U.S.A.). To determine intracellular cytokines, SMCs were cultivated in 24-well plates (Costar) with Brefeldin A (10 μg/mL, Sigma) for 24 h. Next, cells were washed with PBS-BSA solution, labeled with fluorochrome-conjugated CD4 or B220. After fixation and 0.5% saponin (Sigma) permeabilization procedure samples were incubated with fluorochrome-conjugated anti-IL-4, IFN-γ, IL-10 and IL-12p40/p70 antibodies, or the respective isotype controls (BD-Pharmingen) were used in all analysis, fixed and stored at 4°C for flow cytometry acquisition.

### Statistical analysis

Values for all measurements are expressed as mean ± SEM. Differences between groups were considered significant when *P *values were < 0.05, using the *Mann-Whitney *test.

## Abbreviations

MatAb: maternal antibodies; d-o: day old; OVA: ovalbumin; Fc*γ*RIIB: IgG Fc receptor; PCA: passive cutaneous anaphylaxis.

## Authors' contributions

JRV carried out all experimental assays, performed the statistical analysis and helped to draft the manuscript, BPM helped to carry out all assays, CAB and EFT helped the acquisition of data in the FACs assays, AEF helped to carry out the cell culture assays, AJSD participate in critically revising the manuscript with important intellectual contribution and MNS developed the study design, the manuscript draft and coordinated the research group. All authors read and approved the final manuscript.

## References

[B1] Jarrett E Hall E Selective Suppression of IgE Antibody Responsiveness by Maternal Influence Nature 1979 280 145 147 10.1038/280145a0 95350

[B2] Jarrett EE Hall E IgE Suppression by Maternal IgG Immunology 1983 48 49 58 6848454 PMC1454004

[B3] Jarrett EE Hall E The Development of IgE-Suppressive Immunocompetence in Young Animals - Influence of Exposure to Antigen in the Presence or Absence of Maternal Immunity Immunology 1984 53 365 373 6490089 PMC1454820

[B4] Roberts SA Turner MW Specific Suppression of Rat Ige Responses with Milk from Immunized Females and with Feeds of Serum Antibody Immunology 1983 48 195 199 6848452 PMC1453993

[B5] Seeger M Thierse HJ Lange H Shaw L Hansen H Lemke H Antigen-independent suppression of the IgE immune response to bee venom phospholipase A(2) by maternally derived monoclonal IgG antibodies Eur J Immunol 1998 28 2124 2130 10.1002/(SICI)1521-4141(199807)28:07<2124::AID-IMMU2124>3.0.CO;2-A 9692881

[B6] Lange H Kiesch B Linden I Otto M Thierse HJ Shaw L Maehnss K Hansen H Lemke H Reversal of the adult IgE high responder phenotype in mice by maternally transferred allergen-specific monoclonal IgG antibodies during a sensitive period in early ontogeny Eur J Immunol 2002 32 3133 3141 10.1002/1521-4141(200211)32:11<3133::AID-IMMU3133>3.0.CO;2-0 12555658

[B7] Fusaro AE Maciel M Victor JR Oliveira CR Duarte AJS Sato MN Influence of maternal murine immunization with Dermatophagoides pteronyssinus extract on the type I hypersensitivity response in offspring Int Arch Allergy Immunol 2002 127 208 216 10.1159/000053865 11979046

[B8] Fusaro AE Brito CA Victor JR Rigato PO Goldoni AL Duarte AJS Sato MN Maternal-fetal interaction: preconception immunization in mice prevents neonatal sensitization induced by allergen exposure during pregnancy and breastfeeding Immunology 2007 122 107 115 10.1111/j.1365-2567.2007.02618.x 17608811 PMC2265981

[B9] Victor JR Fusaro AE Duarte AJD Sato MN Preconception maternal immunization to dust mite inhibits the type I hypersensitivity response of offspring J Allergy Clin Immunol 2003 111 269 277 10.1067/mai.2003.39 12589344

[B10] Hamada K Suzaki Y Leme A Ito T Miyamoto K Kobzik L Kimura H Exposure of pregnant mice to an air pollutant aerosol increases asthma susceptibility in offspring J Toxicol Environ Health, Part a 2007 70 688 695 10.1080/15287390600974692 17365623

[B11] Siegrist CA Neonatal and early life vaccinology Vaccine 2001 19 3331 3346 10.1016/S0264-410X(01)00028-7 11348697

[B12] Rigato PO Fusaro AE Victor JR Sato MN Maternal immunization to modulate the development of allergic response and pathogen infections Immunotherapy 2009 1 141 156 10.2217/1750743X.1.1.141 20635979

[B13] Verhasselt V Milcent V Cazareth J Kanda A Fleury S Dombrowicz D Glaichenhaus N Julia V Breast milk-mediated transfer of an antigen induces tolerance and protection from allergic asthma Nat Med 2008 14 170 175 10.1038/nm1718 18223654

[B14] Vakil M Kearney JF Functional-Characterization of Monoclonal Auto-Antiidiotype Antibodies Isolated from the Early B-Cell Repertoire of Balb/C Mice Eur J Immunol 1986 16 1151 1158 10.1002/eji.1830160920 2428627

[B15] Bogen B Dembic Z Weiss S Clonal Deletion of Specific Thymocytes by an Immunoglobulin Idiotype Embo J 1993 12 357 363 8428591 10.1002/j.1460-2075.1993.tb05664.x PMC413213

[B16] Hiernaux J Bona C Baker PJ Neonatal Treatment with Low-Doses of Anti-Idiotypic Antibody Leads to the Expression of a Silent Clone J Exp Med 1981 153 1004 1008 10.1084/jem.153.4.1004 7019374 PMC2186123

[B17] Borghesi C Nicoletti C Autologous anti-idiotypic antibody response is regulated by the level of circulating complementary idiotype Immunology 1996 89 172 177 10.1046/j.1365-2567.1996.d01-724.x 8943710 PMC1456490

[B18] Heyman B Feedback regulation by IgG antibodies Immunol Lett 2003 88 157 161 10.1016/S0165-2478(03)00078-6 12880686

[B19] Amigorena S Salamero J Davoust J Fridman WH Bonnerot C Tyrosine-Containing Motif That Transduces Cell Activation Signals Also Determines Internalization and Antigen Presentation Via Type-III Receptors for IgG Nature 1992 358 337 341 10.1038/358337a0 1386408

[B20] Muta T Kurosaki T Misulovin Z Sanchez M Nussenzweig MC Ravetch JV A 13-Amino-Acid Motif in the Cytoplasmic Domain of Fc-Gamma-Riib Modulates B-Cell Receptor Signaling Nature 1994 369 340 8183374 10.1038/369340a0

[B21] Brauweiler AM Cambier JC Fc gamma RIIB activation leads to inhibition of signalling by independently ligated receptors Biochem Soc Trans 2003 31 281 285 10.1042/BST0310281 12546702

[B22] Xiang Z Cutler AJ Brownlie RJ Fairfax K Lawlor KE Severinson E Walker EU Manz RA Tarlinton DM Smith KGC Fc gamma RIIb controls bone marrow plasma cell persistence and apoptosis Nat Immunol 2007 8 419 429 10.1038/ni1440 17322888

[B23] Bolland S Ravetch JV Spontaneous autoimmune disease in Fc gamma RIIB-deficient mice results from strain-specific epistasis Immunity 2000 13 277 285 10.1016/S1074-7613(00)00027-3 10981970

[B24] McGaha TL Karlsson MCI Ravetch JV Fc gamma RIIB deficiency leads to autoimmunity and a defective response to apoptosis in Mrl-MpJ mice J Immunol 2008 180 5670 5679 18390752 10.4049/jimmunol.180.8.5670

[B25] Niu HT Sobel ES Morel L Defective B-cell response to T-dependent immunization in lupus-prone mice Eur J Immunol 2008 38 3028 3040 10.1002/eji.200838417 18924209 PMC2828936

[B26] Manetti R Parronchi P Giudizi MG Piccinni MP Maggi E Trinchieri G Romagnani S Natural killer cell stimulatory factor (interleukin 12 [IL-12]) induces T helper type 1 (Th1)-specific immune responses and inhibits the development of IL-4-producing Th cells J Exp Med 1993 177 1199 1204 10.1084/jem.177.4.1199 8096238 PMC2190961

[B27] Rudge EU Cutler AJ Pritchard NR Smith KGC Interleukin 4 reduces expression of inhibitory receptors on B cells and abolishes CD22 and Fc gamma RII-mediated B cell suppression J Exp Med 2002 195 1079 1085 10.1084/jem.20011435 11956299 PMC2193690

[B28] Zhang Y Liu SX Liu J Zhang T Shen Q Yu YZ Ca XT Immune Complex/Ig Negatively Regulate TLR4-Triggered Inflammatory Response in Macrophages through Fc gamma RIIb-Dependent PGE(2) Production J Immunol 2009 182 554 562 19109188 10.4049/jimmunol.182.1.554

[B29] Aluvihare VR Kallikourdis M Betz AG Regulatory T cells mediate maternal tolerance to the fetus Nat Immunol 2004 5 266 271 10.1038/ni1037 14758358

[B30] Guleria I Sayegh MH Maternal acceptance of the fetus: True human tolerance J Immunol 2007 178 3345 3351 17339426 10.4049/jimmunol.178.6.3345

[B31] Munn DH Zhou M Attwood JT Bondarev I Conway SJ Marshall B Brown C Mellor AL Prevention of allogeneic fetal rejection by tryptophan catabolism Science 1998 281 1191 1193 10.1126/science.281.5380.1191 9712583

[B32] Xu CG Mao DL Holers VM Palanca B Cheng AM Molina H A critical role for murine complement regulator Crry in fetomaternal tolerance Science 2000 287 498 501 10.1126/science.287.5452.498 10642554

[B33] Hubeau C Kobzik L Early asthma susceptibility and maternal cytokine imbalance during pregnancy J Immunol 2006 176 S290 S291

[B34] Yamashita T Freigang S Eberle C Pattison J Gupta S Napoli C Palinski W Maternal immunization program spostnatal immune responses and reduces atherosclerosis in offspring Circ Res 2006 99 7 e51 64 10.1161/01.RES.0000244003.08127.cc 16946133

[B35] Zhu XP Meng G Dickinson BL Li XT Mizoguchi E Miao LL Wang YS Robert C Wu BY Smith PD Lencer WI Blumberg RS MHC class I-related neonatal Fc receptor for IgG is functionally expressed in monocytes, intestinal macrophages, and dendritic cells J Immunol 2001 166 3266 3276 11207281 10.4049/jimmunol.166.5.3266 PMC2827247

[B36] Malefyt RD Abrams J Bennett B Figdor CG Devries JE Interleukin-10 (IL-10) Inhibits Cytokine Synthesis by Human Monocytes - an Autoregulatory Role of Il-10 Produced by Monocytes J Exp Med 1991 174 1209 1220 10.1084/jem.174.5.1209 1940799 PMC2119001

[B37] Mota I Wong D Homologous and Heterologous Passive Cutaneous Anaphylactic Activity of Mouse Antisera During Course of Immunization Life Sciences 1969 8 813 10.1016/0024-3205(69)90099-X 5306608

